# The endocytic receptor protein LRP-1 modulate P-glycoprotein mediated drug resistance in MCF-7 cells

**DOI:** 10.1371/journal.pone.0285834

**Published:** 2023-09-28

**Authors:** Aubery Henry, Marine Mauperin, Jerome Devy, Stephane Dedieu, Lise Chazee, Cathy Hachet, Christine Terryn, Laurent Duca, Laurent Martiny, Emmanuelle Devarenne-Charpentier, Hassan El Btaouri

**Affiliations:** 1 UMR-CNRS 7369 Matrice Extracellulaire et Dynamique Cellulaire (MEDyC), UFR SEN, URCA, Reims cedex, France; 2 Technical Platform for Cellular and Tissue Imaging (PICT), UFR Pharmacie, URCA, Reims, France; China Medical University (Taiwan), TAIWAN

## Abstract

Multidrug resistance (MDR) is a major obstacle to successful cancer chemotherapy. A typical form of MDR is due to the overexpression of membrane transport proteins., such as Glycoprotein-P (P-gp), resulting in an increased drug efflux preventing drug cytotoxicity. P-gp is mainly localized on the plasma membrane; however, it can also be endocytosed resulting in the trafficking of P-gp in endoplasmic reticulum, Golgi, endosomes, and lysosomes. The lysosomal P-gp has been found to be capable of transporting and sequestering P-gp substrates (e.g., Doxorubicin (Dox)) into lysosomes to protect cells against cytotoxic drugs. Many translational studies have shown that low-density lipoprotein receptor-related protein-1 (LRP-1) is involved in endocytosis and regulation of signalling pathways. LRP-1 mediates the endocytosis of a diverse set of extracellular ligands that play important roles in tumor progression. Here, we investigated the involvement of LRP-1 in P-gp expression and subcellular redistribution from the cell surface to the lysosomal membrane by endocytosis and its potential implication in P-gp-mediated multidrug resistance in MCF-7 cells. Our results showed that MCF-7 resistant cells (MCF-7R) overexpressed the P-gp, LRP-1 and LAMP-1 and were 11.66-fold resistant to Dox. Our study also revealed that in MCF-7R cells, lysosomes were predominantly high density compared to sensitized cells and P-gp was localized in the plasma membrane and lysosomes. LRP-1 blockade reduced lysosomes density and level of LAMP-1 and P-gp. It also affected the subcellular distribution of P-gp. Under these conditions, we restored Dox nuclear uptake and ERK 1/2 activation thus leading to MCF-7R cell sensitization to Dox. Our data suggest that LRP-1 is able to modulate the P-gp expression and subcellular redistribution by endocytosis and to potentiate the P-gp-acquired Dox resistance.

## 1. Introduction

Drug resistance is an obstacle that impairs the success of cancer therapies. This resistance occurs after repeated cycles of chemotherapy leading to the acquisition of tumor resistance [1]. Multiple mechanisms contribute to drug resistance, such as increased drug efflux, altered drug metabolism and activation of downstream transduction pathways [2, 3]. Recent studies have suggested that immune cells in tumor microenvironment (TME) play important roles in mediating acquired drug resistance [4, 5]. Indeed, reduced number of cytotoxic effector immune cells within the TME drive the tumor-mediated immunosuppression and could trigger acquired resistance development against immune checkpoint inhibitors [6–8]. Additionally, it has also been reported that the extracellular matrix influences tumor chemoresistance [9]. In fact, In fact, several studies confirmed the implication of collagen and fibronectin in chemoresistance induction [10–12]. Our previous study demonstrated that Thrombospondin-1 contribute to the modulation of P-gp drug resistance in thyroid carcinoma FTC-133 cells [13].

The main mechanism of cell drug resistance involves the ABC (ATP-binding cassette) protein transporters which pump drug molecules out of cells [14]. ABC transporters include the multidrug resistance (MDR) protein or P-glycoprotein (MDR1/P-gp/ABCB1), the multidrug resistance-associated proteins (MRP1, ABCC1) and the breast cancer resistance proteins (BCRP, ABCG2) [15, 16]. P-gp, a 170-kDa membrane protein, is localized not only at the plasma membrane but also in intracellular compartments such as endoplasmic reticulum and Golgi [17–19]. Several studies have suggested that P-gp, when present at the plasma membrane, functions as a pump to efflux drug out the cell, but also, when localised at the lysosomal membrane, can play another functional role inducing drug resistance. In this case, lysosomal membrane P-gp transports substrates into the organelle, thus preventing drugs effects. [20]. These results suggested that there was an intracellular pool of P-gp that originated from the plasma membrane by an endocytosis pathway. Several studies have focused on the function of P-gp and ways to overcome P-gp-mediated MDR [2, 14, 21, 22]. However, little is known about the processes of cellular distribution of P-gp and its intracellular traffic.

The LDL (low-density lipoprotein) receptor family is a group of transmembrane proteins that bind a variety of ligands. Currently identified LRP-1 ligands include proteases, protease inhibitor complexes, extracellular matrix proteins, growth factors, toxins, and viral proteins [23–26]. LRP-1 (LDL receptor-related protein 1) is an important member of this family with multiple ligand binding sites and is expressed in several cell types. [27–29]. LRP-1 is synthesized as a precursor glycoprotein of 600 kDa and cleaved into two non-covalently associated subunits (α, 515 kDa and β, an 85 kDa). The endocytosis and intracellular trafficking of LRP-1 plays a key role in regulating the cellular functions and activities of several receptors and plasma membrane proteins that molecularly interact with LRP-1, such as platelet-derived growth factor receptor β (PDGFR β), urokinase-plasminogen activator receptor (uPAR) and β1-integrin [30–33]. The broad ligands that bind LRP-1 enable this receptor to serve as a sensor of the cellular microenvironment and regulator of cell signalling and cell physiology in response to numerous extracellular stimuli [32, 34]. LRP-1 also regulates cell signalling by trafficking receptor-ligand complexes into endosomes and consequently by controlling the abundance of these receptors at the plasma membrane [35].

Previous work showed that LRP-1 promoted invasion, survival or metastatic dissemination of thyroid carcinoma [36] and breast cancer cells [37–39]. Other studies established that LRP-1 may constitute a strong predictive marker of clinical outcome in 12 types of cancers [40].

With the purpose of establishing a functional relationship between LRP-1-mediated endocytosis and P-gp acquired-resistance, here we explored the ability of LRP-1 to modulate P-gp-induced resistance to Dox in MCF-7 cells.

## 2. Materials and methods

### 2.1. Materials

MCF-7, a human breast carcinoma cell line derived from the pleural effusion metastasis, weas purchased from ATCC (USA). Dox was purchased from Farmitalia (Italy). MCF-7 resistant cells (MCF-7R) were obtained from MCF-7 parental cells by increased Dox treatment. DMEM/F-12, trypsin and Lipofectamine RNAiMAX were from Invitrogen (France). Bovine fetal serum was from Dutscher (France). LRP-1 polyclonal antibody *‘ab92544’* was from Abcam (France). Caspase-7 ‘*#9492’*, ERK 1/2 ‘*#4695’*, Phospho-ERK 1/2 ‘*#4370’*, LAMP-1 ‘*#9091’* and P-gp ‘*#13342’* antibodies were from Cell Signaling Technology (France). LRP-1 siRNA kit ‘*sc-40101’* was purchased from Santa Cruz Biotechnology (USA). Kit ECL was from Amersham (Germany). U0216, a elective inhibitor of MEK-1 and MEK-2, was from Cell Signaling Technology (France). UptiBlue and BCA kit ‘23225’ were from Uptima (Thermofisher, France). CaspACE assay kit ‘*G8091*’ was from Promega (France). β-actin antibody ‘*A5316*’, invertase enzyme ‘I9274’ and all other reagents were from Sigma (USA). Histidine-tagged RAP was purified as previously described [41] and used as an antagonist of LRP-1-dependent endocytosis [41, 42].

### 2.2. Cell culture

MCF-7R cells were selected from MCF-7 parental cells by gradual increase of the Dox concentration (from 10 to 800 nM) according to protocol of Chen et al. [43] modified. MCF-7 and MCF-7R cells were cultured in 75 cm^2^ flasks at 37°C containing DMEM/F-12 (1:1) supplemented with 10% (v/v) foetal calf serum, streptomycin (100 μg/ml) and penicillin (100 IU/ml) in a 5% CO_2_. Cells were then trypsined and cultured in appropriate well plates for cell viability, flow cytometry, spectrofluorometry, Western blot analysis, mRNA extraction and caspase assay.

### 2.3. Cell viability determination

Cells plated in 96-well plates at 10^4^ cells/mL were incubated until they reached the exponential phase of growth. The medium was then substituted with serum free medium with or without different Dox concentrations (from 0 to 10 μM), in presence or absence of RAP (500 nM) or U0126 (1 μM). After 48 h and 72h incubation, 10% (v/v) UptiBlue was added, and the cells incubated for 3 additional hours. The viability was determined by spectrofluorometry (λex: 530–560 nm; λem: 590 nm). The results were calculated as percentage of control as follows: (% of treated viable cells versus untreated viable cells).

### 2.4. Nuclear incorporation of Dox

Cells plated in Petri dish at 10^4^ cells/mL were incubated with Dox 1 μM in presence or absence of RAP (500 nM) for 5 hours and placed in the medium without phenol red. The nuclear Dox incorporation was measured by spectrofluorometry over the wavelength range 500–700 nm [44]. The nuclear Dox was semi-quantified by the fluorescence emission intensity of the band at 690 nm (within the nuclear perimeter of 50 nuclei) optical fields. The data of 5 different random was analysed by Labspec software (Horiba).

### 2.5. Western blot

Cells were centrifuged at 3000 g for 5 min (4°C) and washed with ice-cold PBS. Cell pellets were lysed by lysis buffer containing 10 mM Tris pH 7.4 (150 mM NaCl, 5 mM EDTA, 1 mM orthovanadate, 1 mM dithiothreitol, 10 μg/mL leupeptin, 10 μg/mL aprotinin, 10% (v/v) glycerol, 1% (v/v) Brij). Cell lysates were placed on ice for 20 min and centrifuged (14,000 g, 15 min, 4°C). The protein concentration was determined by BCA assay kit following the manufacturer’s instructions of Thermofisher scientific ‘*2161296’*. Proteins were separated using SDS-PAGE analytical technique according to the Bio-rad PROTEAN specification. In brief, 20–25 μg of protein sample were mixed with 4X sample loading buffer and heated at 70°C for 10 min. Samples were then loaded on the gel (10% polyacrylamide). Electrophoresis was performed at room temperature for approximately 45 min using a constant voltage (200V) in 1X solution of SDS running buffer. The proteins were then transferred to nitrocellulose sheets for approximately 125V/60 min using the Trans-Blot® Cell system according to the Bio-rad manufacturer’s instructions. The nitrocellulose sheets were then probed with the appropriate antibodies (polyclonal anti-LRP-1: dilution 1/1000; polyclonal anti-caspase-7: dilution 1/1000; polyclonal anti-P-gp: dilution 1/1000; polyclonal anti-PhosphoERK1/2: dilution 1/1000; polyclonal anti-ERK1/2: dilution 1/1000; polyclonal anti-LAMP-1: dilution 1/1000; monoclonal anti-β-actin: dilution 1/8000). Horseradish peroxidase-conjugated IgG were used as secondary antibodies (dilution 1/4000 and 1/10,000 respectively) and the detection was realised by enhanced chemiluminescence kit.

### 2.6. Caspase-3/7 activity

Cells were washed twice with PBS and scrapped with ice-cold lysis buffer. Caspase-3/7 activity was measured by incubating 50 μg of cytosolic fraction with a colorimetric substrate and absorbance was measured at 405 nm according to the Promega manufacturer’s instructions ‘TB323’.

### 2.7. Cell transfection

Cell transfection was realized using Lipofectamine RNAiMAX following the manufacturer’s instructions of Invitrogen ‘MAN0007825 Rev. 1.0. Briefly, MCF-7 cells were incubated for 1–3 days at 37°C with either non-targeting control scRNA (scRNA-MCF-7R cells) or siRNA specific to LRP-1 (siRNA- MCF-7R cells) and Lipofectamine RNAiMAX Reagent (1:1 ratio).

### 2.8. Immunofluorescence and internal vesicles labelling

MCF-7R cells, scRNA-MCF-7R cells and siRNA-MCF-7R cells were plated onto 1% gelatin-coated glass slides for 24 h at 37°C. MCF-7R cells were then incubated in presence or absence of RAP (500 nM) for 12h. Cells were fixed in phosphate buffered saline (PBS) containing 4% paraformaldehyde for 15 min at room temperature and incubated for 1 h in PBS containing 1% bovine serum albumin and overnight at 4°C with primary antibodies raised against LRP-1 (1/500) or LAMP-1 (1/600). Then, the slides were washed five times with ice-cold PBS and incubated with secondary antibodies conjugated to Alexa Fluor 488 (1/500) or Alexa Fluor 568 (1/500) for 2 h at room temperature. Confocal microscopy images of fixed cells were collected with a Zeiss (Oberkochen, Germany) LSM710 Meta confocal microscope using either a ×63 Plan Apochromat objective (oil immersion, 1.40 NA, DIC) at a 132 nm.pixel-1 resolution, leading to a slight XY oversampling. The Zen software program was used to acquire images as previously detailed in [41].

### 2.9. Isolation of endocytic organelles by density gradient centrifugation

Following incubation in presence or absence of RAP (500 nM) for 12h, 2.10^6^ cells were washed three times with ice-cold PBS and suspended in homogenization buffer for isolation of endocytic organelles using a gradient centrifugation as described by de Araujo et al. (2015) [45]. After centrifugation, aliquots (1 ml) were collected and the sucrose gradient was analysed using invertase enzyme assay as described by Gusakov et al (2011) [46]. Aliquots were conserved at -80°C.

### 2.10. Sucrose assay

Sucrose in aliquot was measured using DNS Assay as described by Gusakov et al (2011) [46]. Briefly, 50 μL of each diluted aliquot (10x) was incubated with 100 μl of acetate buffer (pH 4.7) and 50 μL of invertase enzyme (100 unit.mL^-1^) for 60 min at 37°C. Then, the DNS reagent (100 μL) was added, and the mixture was incubated in a boiling water bath for 5 min. After cooling to room temperature, the absorbance of the supernatant at 540 nm was measured.

### 2.11. Statistics

Each experiment was realized at least in triplicate with three independent cultured cells. Data were presented as mean ± SEM. The statistical calculation was based on the Student’s t-test. p values referring to corresponding control are ** and ¥¥ p<0.01, *** and ¥¥¥ p<0.001, **** and ¥¥¥¥ p<0.0001.

## 3. Results

To study LRP-1 effects on multidrug resistant cells, two human breast carcinoma cells were used: parental MCF-7 cells sensitive to Dox called MCF-7S and MCF-7 cells resistant to Dox that were obtained by stepwise exposure of MCF-7S cells to increasing doses of Dox and called MCF-7R [47]. No significant difference in the morphology and growth rates between the two-line cells MCF-7S and MCF-7R cells was observed.

**Fig 1:**
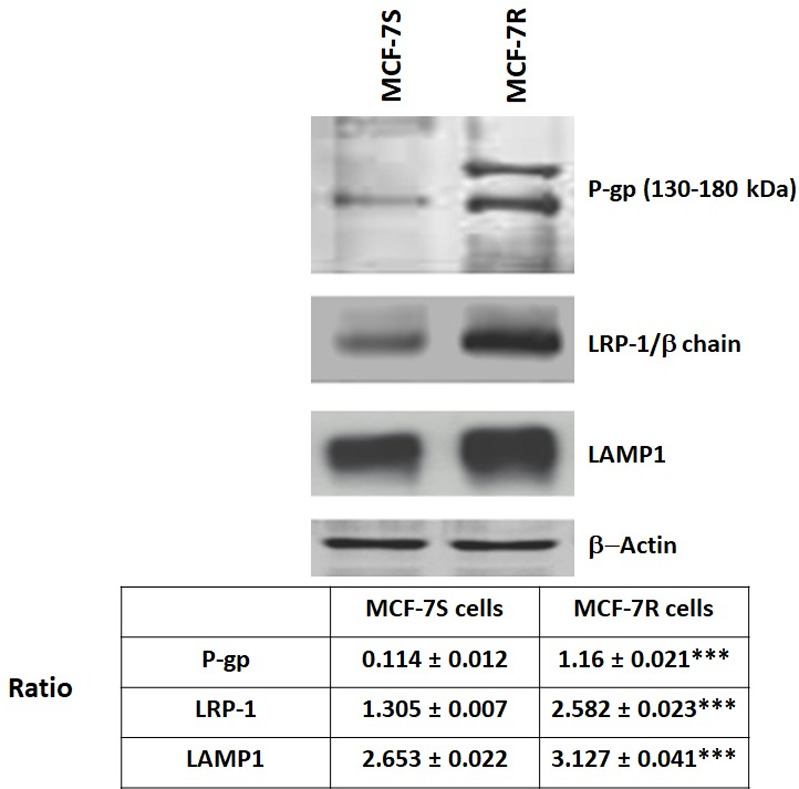
Overexpression of P-gp and LRP-1 in MCF-7R. MCF-7S and MCF-7R cells were cultured for 24 hours. Detection of P-gp, Lamp-1 and LRP-1/β chain was evaluated by Western-blot. β-actin antibody was used as a control. The results were represented by three independent experiments. Quantity one software was used to quantify the intensity of the bands. Student’s t-test was used for the statistical significance of different values. *** p<0.001 for MCF-7R cells compared to MCF-7S cells. The ratio was calculated with densitometry value of the protein of interest/ densitometry value of the β-actin.

We first analysed the expression of P-gp and LRP-1 in both cell types and Western blot analysis clearly showed a higher level of P-gp and LRP-1 expression in MCF-7R cells compared to MCF-7S cells (Fig 1).

**Fig 2:**
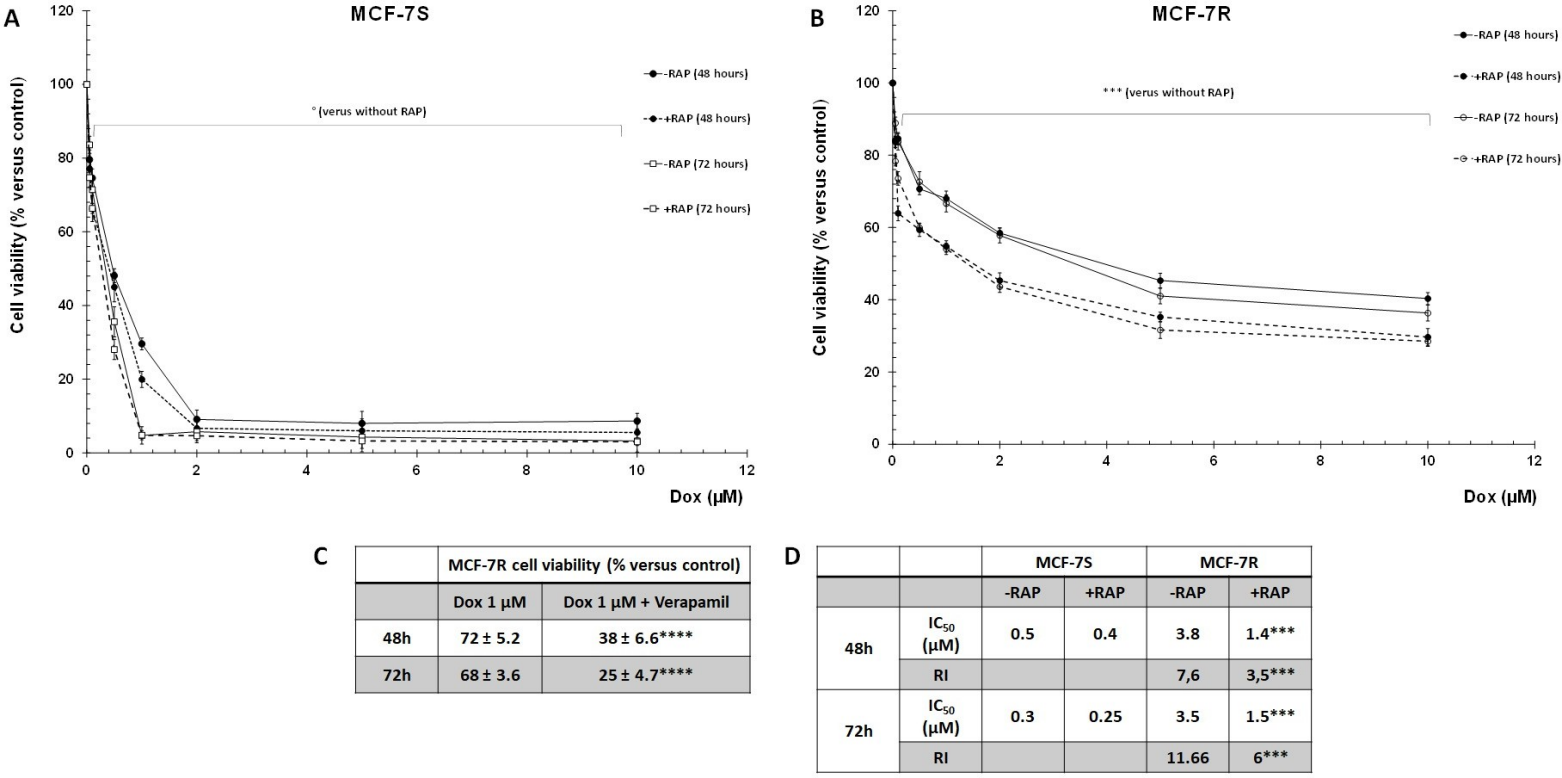
RAP sensitized MCF-7R cells to Dox cytotoxic effects and reduced IC_50_. **A and B**, MCF-7S and MCF-7R cells were treated with different Dox concentrations (from 0 to 10 μM) with or without 500 nM RAP. After 48 and 72h, cell viability was measured by UptiBlue Viable Cell Counting Assay. The results were presented as percentage of control and represented with standard deviation (S.D.) of at least three independent experiments. Student’s t-test was used for the statistical significance of different values.° NS, *** p<0.001 compared to cells cultured without RAP. **C**, MCF-7R cells were pretreated with Verapamil (5 μM) for 6h and incubated with Dox (1 μM). After 48 and 72h, cell viability was measured using UptiBlue Viable Cell Assay. The results obtained from three independent experiments (% of control) were represented with standard deviation (S.D.). Student’s t-test was used for the statistical significance of different values. **** p<0.0001 for Verapamil and Dox-MCF-7R cells compared to Dox-MCF-7R cells. **D**, summary table of IC_50_ and RI (resistance Index). IC_50_ represents the mean half maximal inhibitory concentration. RI was assessed using the quotient of the IC_50_ values (IC_50_ MCF-7R/IC_50_ MCF-7) in each treatment conditions. Student’s t-test was used for the statistical significance of different values. *** p<0.001 for RAP-MCF-7R cells compared to untreated MCF-7R cells.

The MCF-7R cell chemosensitivity to Dox was compared to that of MCF-7S cells by cell incubation with increasing Dox concentrations with or without 500 nM RAP, a universal antagonist of ligand binding to LRP-1 (Fig 2). As expected, Dox decreased MCF-7S cell viability in a dose-dependent manner with an IC_50_ = 0.5 and 0.3 μM for a period of 48 and 72h respectively (Fig 2A). In contrast, MCF-7R cells exhibited a lower sensitivity to Dox, confirmed by an increase of IC_50_ to 3.8 and 3.5 μM for a period of 48 and 72h respectively and an increase of RI (resistance index = ratio IC_50_ MCF-7R/IC_50_ MCF-7S) to 7.6 and 11.66 for a period of 48 and 72h respectively (Fig 2B and 2D). The P-gp mediated Dox resistance was confirmed by Verapamil (P-gp inhibitor), indeed, Verapamil (5 μM) pre-treatement of MCF-7R cells sensitized cells to Dox cytotoxic effects (Fig 2C). Moreover, treatment of MCF-7R cells with RAP induced an increase of Dox cytotoxicity confirmed by lower IC_50_ (1.4 and 1.5 μM for a period of 48 and 72h respectively) and RI (Fig 2D). These results clearly demonstrated that RAP partially restored the MCF-7R cell sensitivity to Dox.

**Fig 3:**
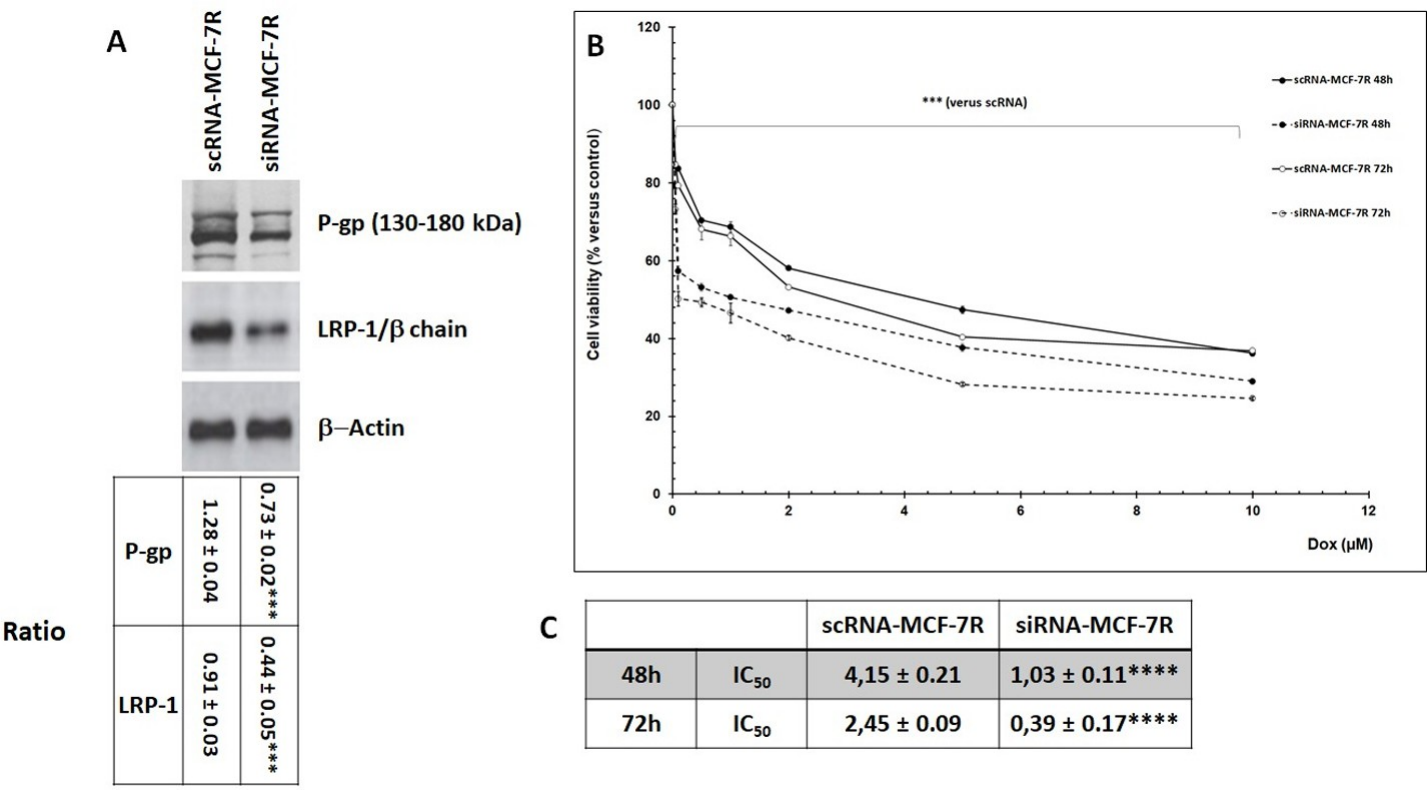
LRP-1 silencing by siRNA reduced P-gp expression and sensitized MCF-7R cells to Dox. **A**, scRNA-MCF-7R and siRNA-MCF-7R cells were cultured for 24 hours. Detection of P-gp and LRP-1/β chain was evaluated by Western-blot. β-actin antibody was used as a control. The intensity of the bands was quantified by densitometry using quantity one program. Student’s t-test was used for the statistical significance of different values. *** p<0.001 for siRNA-MCF-7R cells compared to scRNA-MCF-7R cells. The ratio was calculated with densitometry value of the protein of interest/ densitometry value of the β-actin. **B**, scRNA-MCF-7 and siRNA-MCF-7R cells were incubated with Dox at concentrations ranging from 0 to 10 μM. After 48 and 72h, cell viability was measured using UptiBlue Viable Cell Assay. The results obtained from three independent experiments (% of control) were represented with standard deviation (S.D.). Student’s t-test was used for the statistical significance of different values. *** p<0.001 for siRNA-MCF-7R cells compared to scRNA-MCF-7R cells. **C**, summary table of IC_50_. IC_50_ represents the mean half maximal inhibitory concentration. Student’s t-test was used for the statistical significance of different values. **** p<0.0001 for siRNA-MCF-7R cells compared to scRNA-MCF-7R cells.

To clarify the role of LRP-1 in cell resistance, LRP-1 expression was silenced by small RNA interference in MCF-7R cells (called siRNA-MCF-7R cells). The efficiency of the transfection was evaluated in MCF-7R cells transfected with scrambled RNA (called scRNA-MCF-7R cells). As shown in Fig 3A, Western blot analysis revealed a decrease of the LRP-1 and P-gp expression in siRNA-MCF-7R cell as compared to that in scRNA-MCF-7R cells.

The chemo-sensitivity of these cells was then evaluated in presence of increasing Dox concentrations at 48 and 72 hours (Fig 3B). The cell viability assays clearly showed that Dox induced a significant decrease of siRNA-MCF7R cell viability and reduced the value of IC_50_ from 4.2 to 1μM and from 2.8 to 0.5μM for a period of 48 and 72 hours respectively (Fig 3C). These results demonstrated that LRP-1 silencing sensitized MCF-7 resistant cells to Dox and confirm the role of LRP-1 in MCF-7 cell chemoresistance.

**Fig 4:**
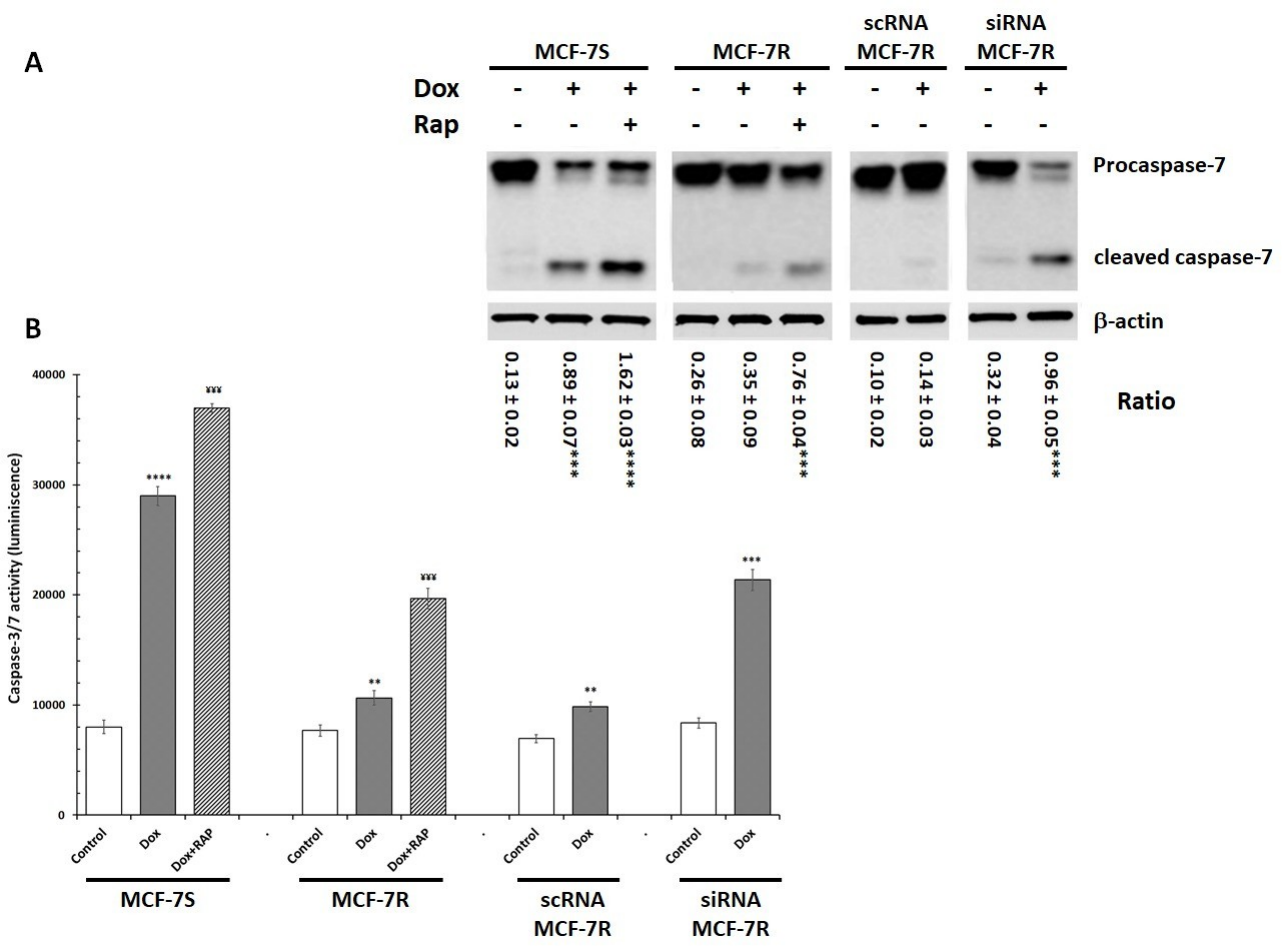
RAP and LRP-1 silencing by siRNA promoted the caspase-7 activation in Dox-treated MCF-7R cells. MCF-7S, MCF-7R, scRNA-MCF-7R and siRNA-MCF-7R cells were incubated with 5 μM Dox with or without 500 nM RAP for 12 hours. A, Procaspase-7 and cleaved caspase-7 were detected by Western blotting. β-actin antibody was used as a control. The results were represented by three independent experiments. Quantity one software was used to quantify the intensity of the bands. Student’s t-test was used for the statistical significance of different values. *** p<0.001 and **** p<0.0001 for treated cells compared to untreated cells. The ratio was calculated with densitometry value of the protein of interest/ densitometry value of the β-actin. **B**, Caspase-7 activity was measured by caspACE assay kit. The results obtained from three independent experiments were represented with standard deviation (S.D.). Student’s t-test was used for the statistical significance of different values. ** p<0.01 and **** p<0.0001 compared to control cells. ¥¥¥ p<0.001 compared to Dox-treated cells.

As previously reported by Rath et al. [48], Dox decreased cell viability by triggering apoptosis in a caspase-dependent manner. However, MCF-7 cells do not express caspase-3, and caspase-7 might compensate for this lack of expression [49]. We thus analysed caspase-7 activation in MCF-7S and MCF-7R cells incubated with or without Dox (1 μM) in presence or in absence of RAP (500 nM) for 12 h. The caspase-7 activity was measured by Western-blot and Caspase-Glo 3/7 assay System (Fig 4). We showed that Dox induced the caspase-7 activation in MCF-7S cells and the presence of RAP amplified this effect. In MCF-7R cells, Dox alone induced a very low activation of caspase-7 compared to MCF-7S cells. However, RAP addition amplified the Dox-induced activation of the caspase-7. This suggests that LRP-1 could be responsible for the low level of caspase-7 activation and the decrease of Dox cytotoxic effect. We then analysed caspase-7 activation in transfected cells and our data showed that both caspase-7 cleavage and activity was significantly increased in siRNA-MCF-7R cells thus confirming that LRP-1 was involved in MCF-7 cell resistance to Dox apoptosis effects (Fig 4).

**Fig 5:**
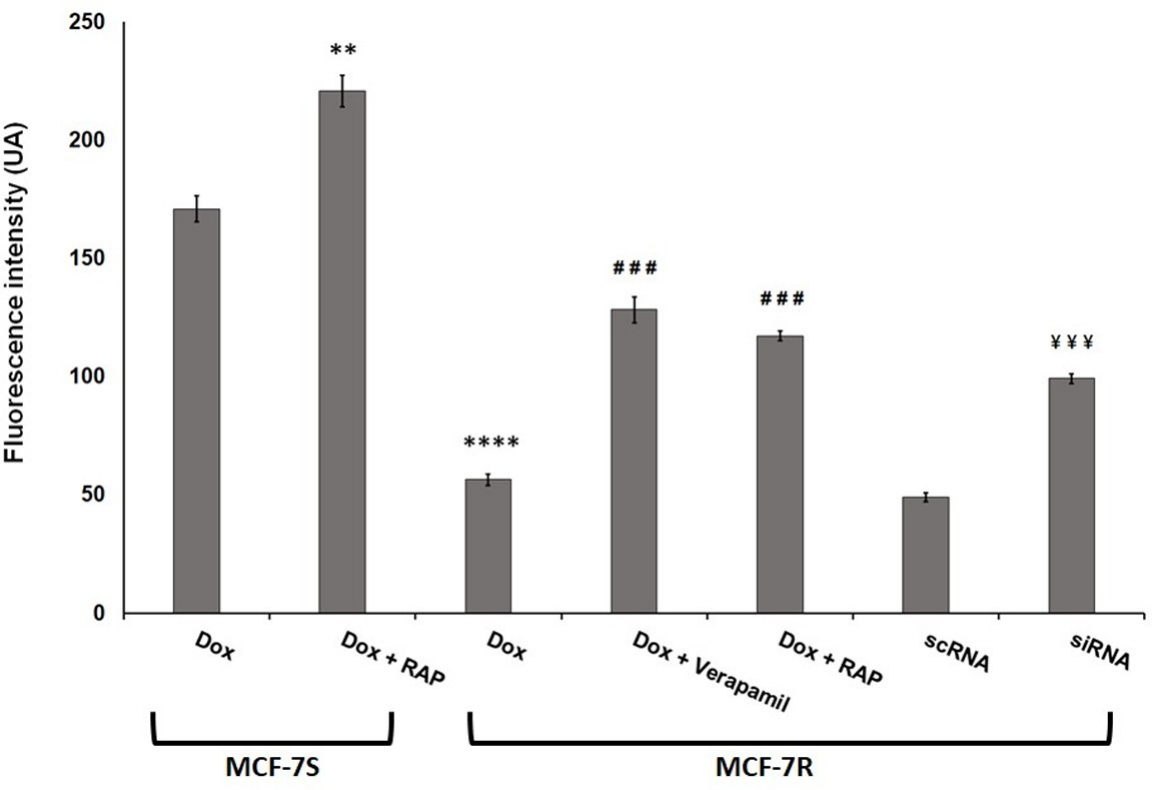
RAP and LRP-1 silencing by siRNA increased Dox nuclear uptake in MCF-7R cells. MCF-7S, MCF-7R, scRNA-MCF-7R and siRNA-MCF-7R cells were placed in petri dishes, pretreated with Verapamil (1 μM) for 6h and with 4 μM Dox with or without 500 nM RAP for 5h. The cells were washed with PBS free of drugs at 4°C. The nuclear Dox was followed through its fluorescence emission spectra by confocal laser microspectrofluorometry. A nuclear spectrum of treated cells was obtained over the wavelength range 500–700 nm [44]. The semi-quantification of nuclear Dox incorporation was evaluated by the fluorescence emission intensity of the band at 590 nm. The results represent mean ± standard deviation (S.D.) of at least three independent experiments. The data was analysed by Labspec software (Horiba). Student’s t-test was used for the statistical significance of different values. **** p < 0.0001 versus Dox-treated MCF-7S cells, ### p < 0.001 versus Dox-treated MCF-7R cells and ¥¥¥ p<0.001 compared to scRNA-cells.

**Fig 6:**
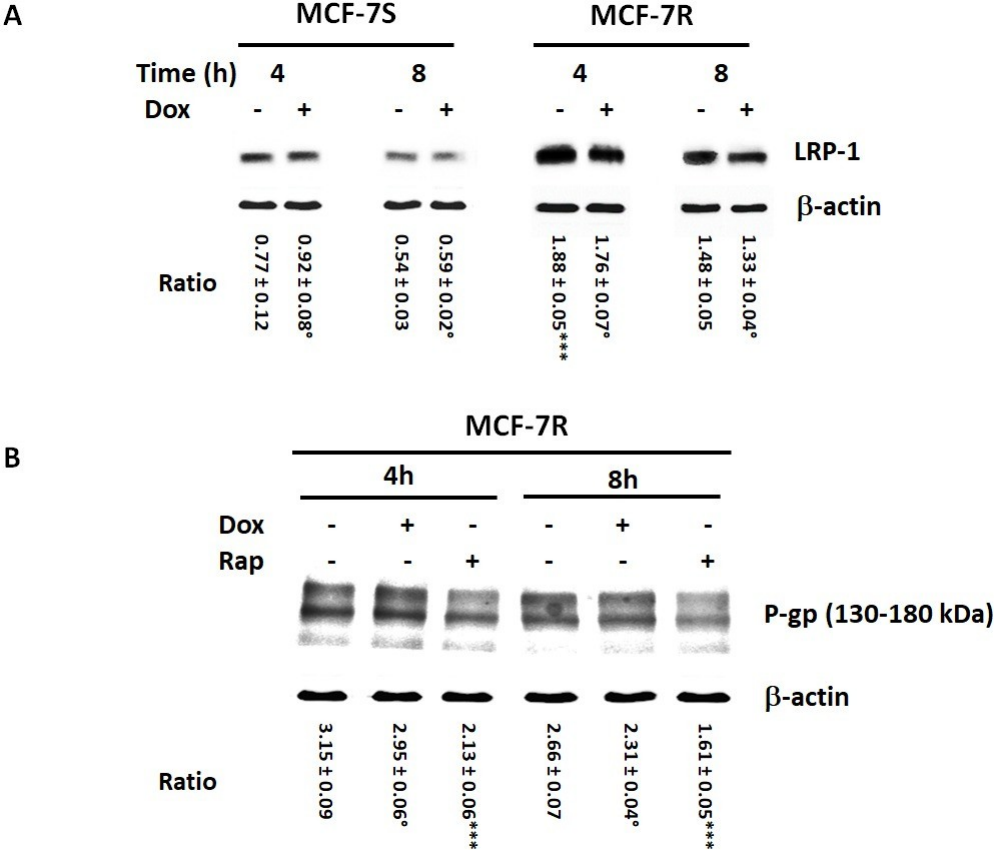
RAP reduced the P-gp expression in MCF-7R cells. **A,** MCF-7S and MCF-7R cells were incubated with or without 5 μM Dox for 4 and 8 hours. Detection of LRP-1/β chain was evaluated by Western-blot. β-actin antibody was used as a control. A representative blot of three independent experiments was shown. The intensity of the bands was quantified by densitometry using quantity one program. Student’s t-test was used for the statistical significance of different values. *** p < 0.001 for MCF-7R versus MCF-7S cells,° NS for Dox-treated cells versus untreated cells. **B,** MCF-7R cells were incubated with or without 500 nM RAP for 4 and 8 hours. Detection of P-gp was evaluated by Western-blot. β-actin antibody was used as a control. A representative blot of three independent experiments was shown. The intensity of the bands was quantified by densitometry using quantity one program. Student’s t-test was used for the statistical significance of different values. *** p < 0.001 for RAP-treated cells versus MCF-7S cells,° NS for Dox-treated cells versus untreated cells. The ratio was calculated with densitometry value of the protein of interest/ densitometry value of the β-actin.

Since the cytotoxic effect of dox is related to its nuclear concentration. we then measured nuclear Dox level in both parental and resistant cells. Fig 5 showed that nuclear Dox level was about 3 times weaker in MCF-7R cells than in MCF-7S cells. The MCF-7R cell pre-treatment by Verapamil (5 μM) increased Dox nuclear uptake about 2.2 times compared MCF-7R treated only by Dox. This result established the P-gp implication in MCF-7R resistance to Dox by decreasing its subcellular accumulation. RAP significantly increased drug nuclear accumulation in MCF-7S as well as in MCF-7R cells and demonstrated that RAP reduced P-gp-induced efflux Dox out of the cell. Moreover, LRP-1 silencing increased drug nuclear accumulation, thus confirming that LRP-1 could modulate P-gp-acquired resistance. We therefore investigated the interrelation between the LRP-1 level and the expression and localization of P-gp in MCF-7S and MCF-7R cells. Thus we analysed LRP-1 and P-gp expression in MCF-7S and MCF-7R cells incubated with or without Dox (1 μM) in presence or in absence of RAP (500 nM). Western blot analysis showed a LRP-1 expression higher in MCF-7R cells than in MCF-7S cells, and its level decreasing with incubation period. Dox had no effects on LRP-1 expression (Fig 6A). The P-gp expression analysis showed that P-gp level was not affected by Dox treatment. However, addition of 500 nM RAP significantly reduced the P-gp expression in MCF-7R cells (Fig 6B). These results confirmed that LRP-1 could modulate the P-gp expression in MCF-7R cells.

**Fig 7:**
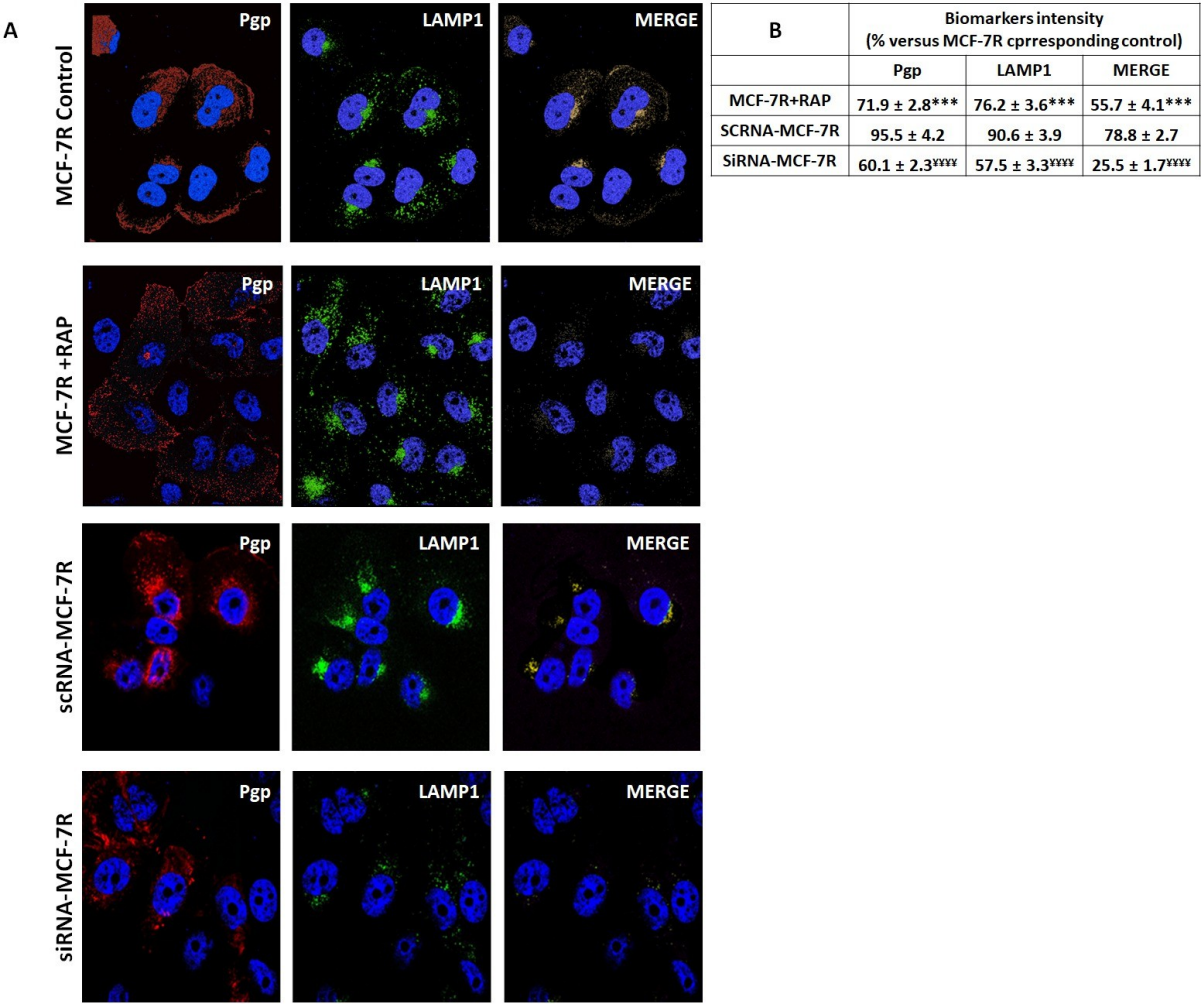
RAP and LRP-1 silencing by siRNA reduced P-gp expression and its distribution in lysosomes of MCF-7R cells. **A**, MCF-7R cells were seeded onto 1% gelatin-coated glass slides for 12 h at 37°C with or without 5 μM Dox in presence or absence of 500 nM RAP for 12 hours. P-gp and Lamp-1 staining respectively with Alexa Fluor 467 and Alexa Fluor 488 were carried out before confocal microscopy analysis as shown in material and methods. The Zen software program was used to acquire images. Isosurface representations were realized using the AMIRA software program. **B,** The biomarker intensity was measured using ImageJ software. For each image, the signal intensity was measured at each cell. The value obtained was the average of the measurements. The results obtained from three independent experiments were represented with standard deviation (S.D.). Student’s t-test was used for the statistical significance of different values. *** p<0.001 compared to MCF-7R control cells. ¥¥¥¥ p<0.0001 compared to scRNA-MCF-7R cells.

P-gp is localized in many intracellular organelles, including endoplasmic reticulum (ER), endosome, lysosome and proteasome [17–19, 50–52]. Furthermore, the endosomal localization is involved in trafficking/recycling P-gp between the cellular pool and the plasma membrane [19]. We thus hypothesised that decreased P-gp expression induced by RAP could result in a decrease of this transporter in the lysosomal compartment. Therefore, we analysed the intracellular localization of P-gp in presence or absence of RAP in MCF-7R cells by confocal immunofluorescence microscopy using anti-LAMP-1 antibody as a lysosome marker (Fig 7). Merged image of P-gp (red) and LAMP-1 (green) revealed distribution of P-gp within lysosome (gold). LRP-1 blockade using RAP reduced P-gp expression and its distribution in lysosomes. This effect was also observed by a siRNA strategy against LRP-1 showing a decrease in P-gp expression and distribution in lysosomes. In these siRNA-MCF-7R cells, we also observed a reduction in the intensity of the fluorescent signal corresponding to the LAMP-1. This result confirmed that LRP-1 affected the localization of P-gp in lysosome and altered lysosomal Dox trapping.

**Fig 8:**
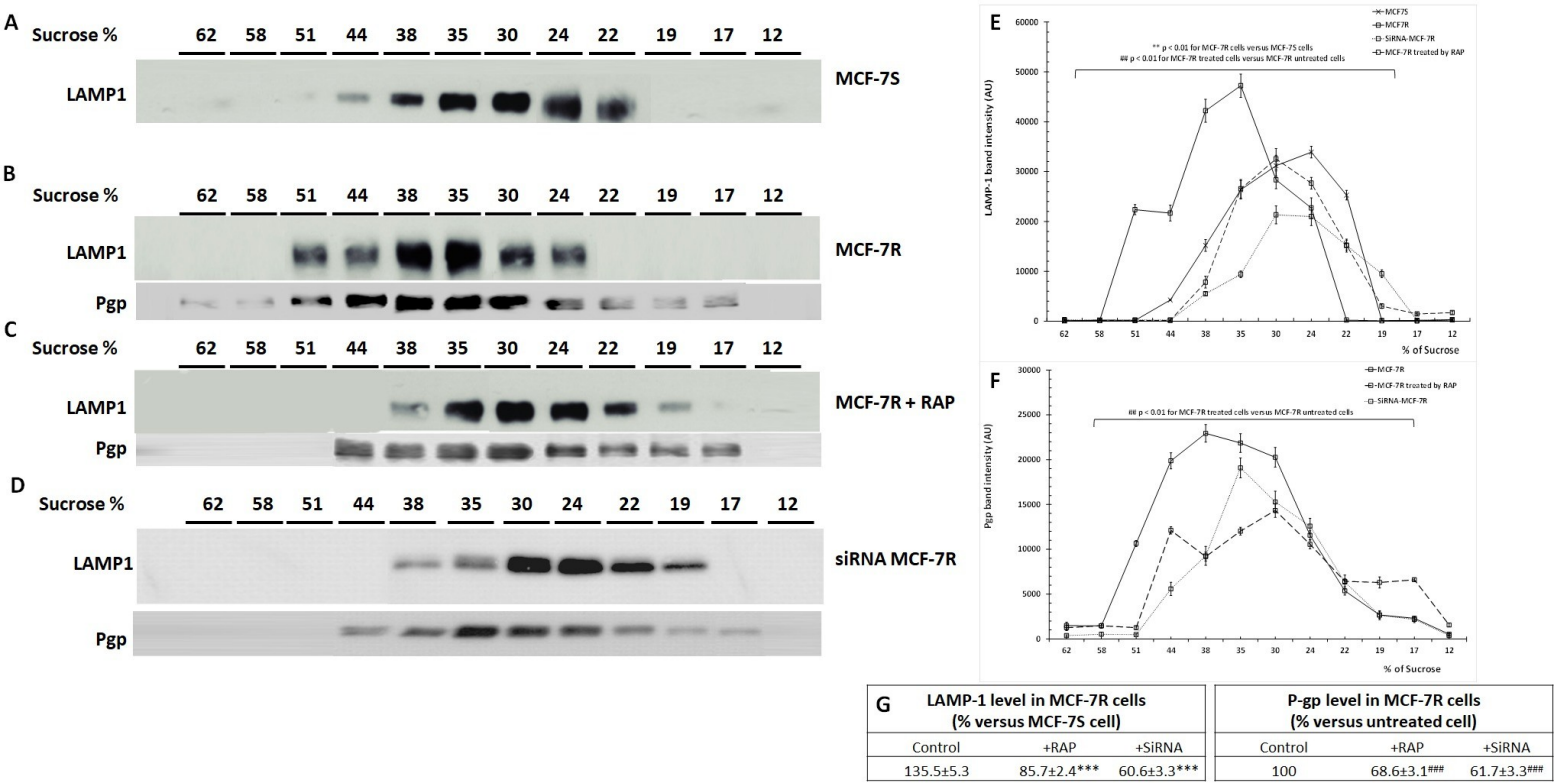
RAP and LRP-1 silencing by siRNA decreased the level and density of lysosome in MCF-7R cells. MCF-7S, MCF-7R cells that incubated with or without 500 nM RAP for 12 hours were used in this experiment. The endocytic organelles were isolated by density gradient centrifugation as detailed in Materials and Methods. sucrose gradient was analysed using invertase enzyme assay as described in Materials and Methods. Detection of P-gp and Lamp-1 were evaluated by Western-blot in all collected aliquots (A-D). The intensity of the bands was quantified by densitometry using quantity one program. Student’s t-test was used for the statistical significance of different values. ** p < 0.01, *** p < 0.001 for MCF-7R cells versus MCF-7S cells, ## p < 0.01, ### p < 0.001 for MCF-7R treated cells versus MCF-7R untreated cells (E,F,G).

To confirm these results, we used density gradient centrifugation to separate intracellular components in particular endocytic organelle. Cell lysate was subjected to sucrose-gradient centrifugation in order to separate the lysososomal fractions from the soluble proteins and disrupted membranes. After centrifugation, we measured sucrose percentage in collected consecutive fractions. The result confirmed that sucrose gradient was maintained between 12 and 62%. Then, we analysed lysosomes and P-gp expression in all collected fractions by Western blot using anti-LAMP-1 and anti-P-gp antibodies respectively (Fig 8). In MCF-7S cells, the fractions 22–44% (of sucrose) contained lysosomes with the majority being concentrated in fraction 30% (Fig 8A). In MCF-7R cells, lysosomes were mainly localized in the fractions 24–51% (of sucrose) with a significant level in the fractions 35–38% of sucrose. This suggested that lysosomes have high density in MCF-7R cells. P-gp detection by Western-blot showed that P-gp displayed a continuous distribution in fractions 17–51% sucrose, peaking in fractions 30–44% corresponding to lysosomal localization (Fig 8B). LRP-1 blockade using RAP or SiRNA specific to LRP-1 significantly decreased the level and density of lysosome in MCF-7R cells. Indeed, LAMP-1 expression was reduced and was essentially localized in the fractions 24–35% (of sucrose) in RAP-treated cells and siRNA-MCF-7R cells. The distribution profile of lysosomes overlapped with that obtained in MCF-7S cells (Fig 8E). We also analysed the LAMP-1 level in all fractions. The results showed that LAMP-1 expression is very high in MCF-7R cells compared to MCF-7S cells. RAP or siRNA treatment of cells reduced the LAMP-1 level in MCF-7R cells (Fig 8G). P-gp expression was also decreased in fractions containing lysosomes after RAP treatment MCF-7R cells. Similar results were obtained in siRNA-MCF-7R cells (Fig 8C and 8D and 8F and 8G).

**Fig 9:**
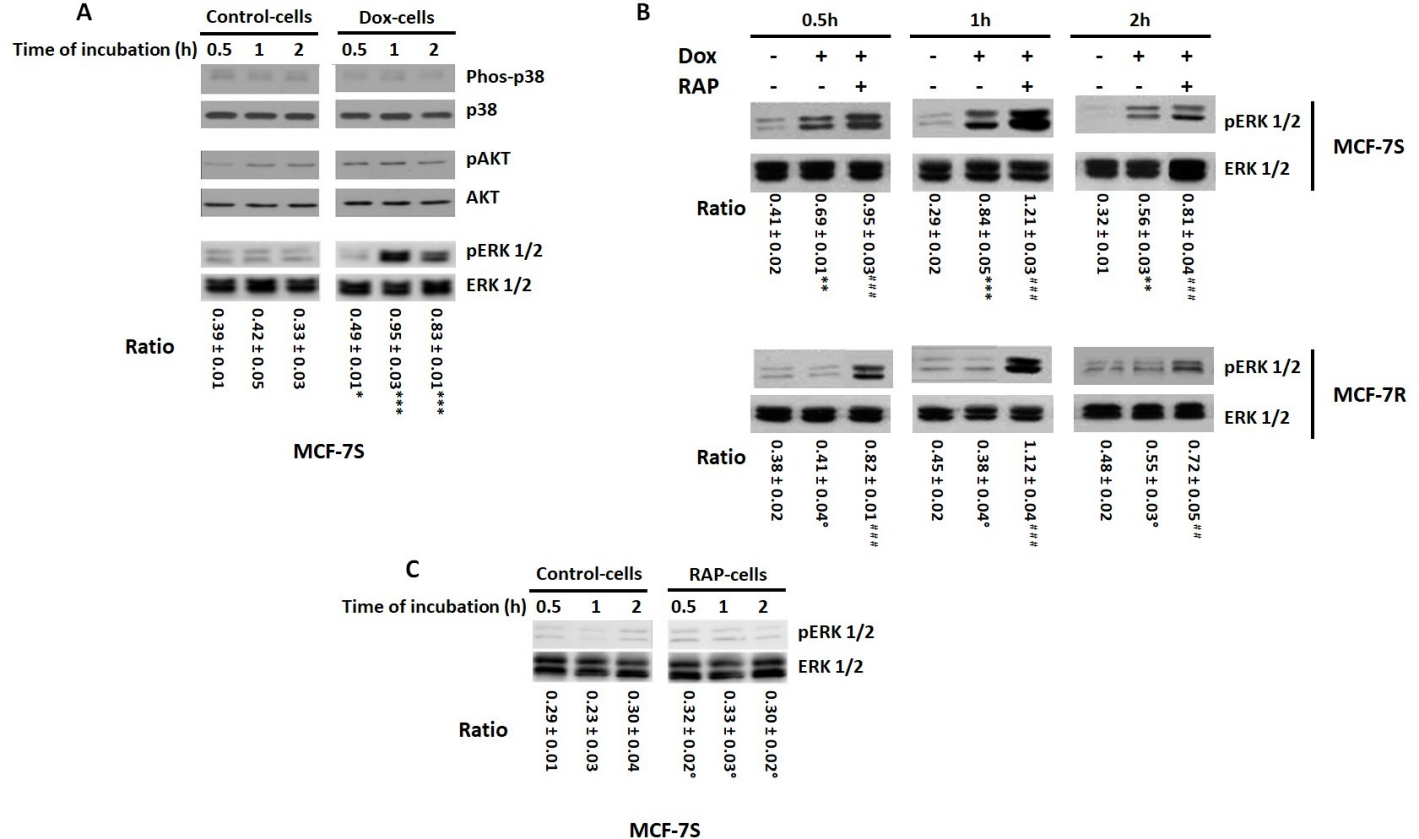
RAP and LRP-1 silencing by siRNA promoted the ERK1/2 phosphorylation in Dox-treated MCF-7R cells. **A**, MCF-7S cells were incubated with 1 μM Dox for 0.5, 1 and 2 hours. Detection of pERK, ERK, pAKT, AKT, Phospho-p38 and p-38 were evaluated by Western-blot. A representative blot of three independent experiments was shown. The intensity of the bands was quantified by densitometry using quantity one program. The ratio was calculated with densitometry value of the pERK/ densitometry value of ERK. Student’s t-test was used for the statistical significance of different values. *** p < 0.001, ** p < 0.01 for Dox treated cells versus untreated cells. **B**, MCF-7S and MCF-7R cells were incubated with 1 μM Dox with or without 500 nM RAP for 0.5, 1 and 2 hours. Detection of pERK and ERK were evaluated by Western-blot. A representative blot of three independent experiments was shown. The intensity of the bands was quantified by densitometry using quantity one program. The ratio was calculated with densitometry value of the pERK/ densitometry value of ERK. Student’s t-test was used for the statistical significance of different values.° NS, ** p < 0.01 and *** p<0.001 for MCF-7 treated cells versus MCF-7S untreated cells, ## p < 0.01 and ### p < 0.001 versus Dox-treated cells. **C**, MCF-7S cells were incubated with or without 500 nM RAP for 0.5, 1 and 2 hours. Detection of pERK and ERK were evaluated by Western-blot. A representative blot of three independent experiments was shown. The intensity of the bands was quantified by densitometry using quantity one program. The ratio was calculated with densitometry value of the pERK/ densitometry value of ERK. Student’s t-test was used for the statistical significance of different values.° NS, RAP treated cells versus untreated cells.

Next, we investigated the signalling pathways involved in cell apoptosis induced by Dox and modulated by LRP-1. Several studies have shown that the p38 MAPK and PI3K/Akt pathways apparently exert a regulatory effect with regard to the induction of cell death; the sustained activation of ERK1/2 appears to be positively related to apoptosis induced by anticancer DNA-damaging drugs in several cell lines [53]. Therefore, we analysed the effects of Dox on p38 and ERK1/2 MAPKs activation in both MCF-7S and MCF7-R cells. The cells were incubated in presence or absence of Dox (1 μM) with or without RAP (500 nM) for 0.5, 1 and 2 hours and phosphorylation of ERK1/2, p38 and AKT was analysed by Western-blot (Fig 9). Results showed that Dox treatment modulated ERK1/2 phosphorylation but had no effect on p38 and AKT (Fig 9A). Indeed, Dox induced ERK1/2 phosphorylation in MCF-7S with a maximum at 1h of time incubation (Fig 9B). This effect was amplified by RAP treatment while RAP alone has no effect on EKR1/2 expression and basal activity (Fig 9C). In MCF7R cells, Dox induced ERK1/2 phosphorylation in the presence of RAP whereas alone had no effect on this MAPK. This result showed that P-gp expression was correlated with a decrease of ERK1/2 phosphorylation and suggested that P-gp-expressed in MCF-7R inhibited the ERK1/2 phosphorylation by Dox. However, RAP treatment restored the Dox-induced ERK1/2 activation.

**Fig 10:**
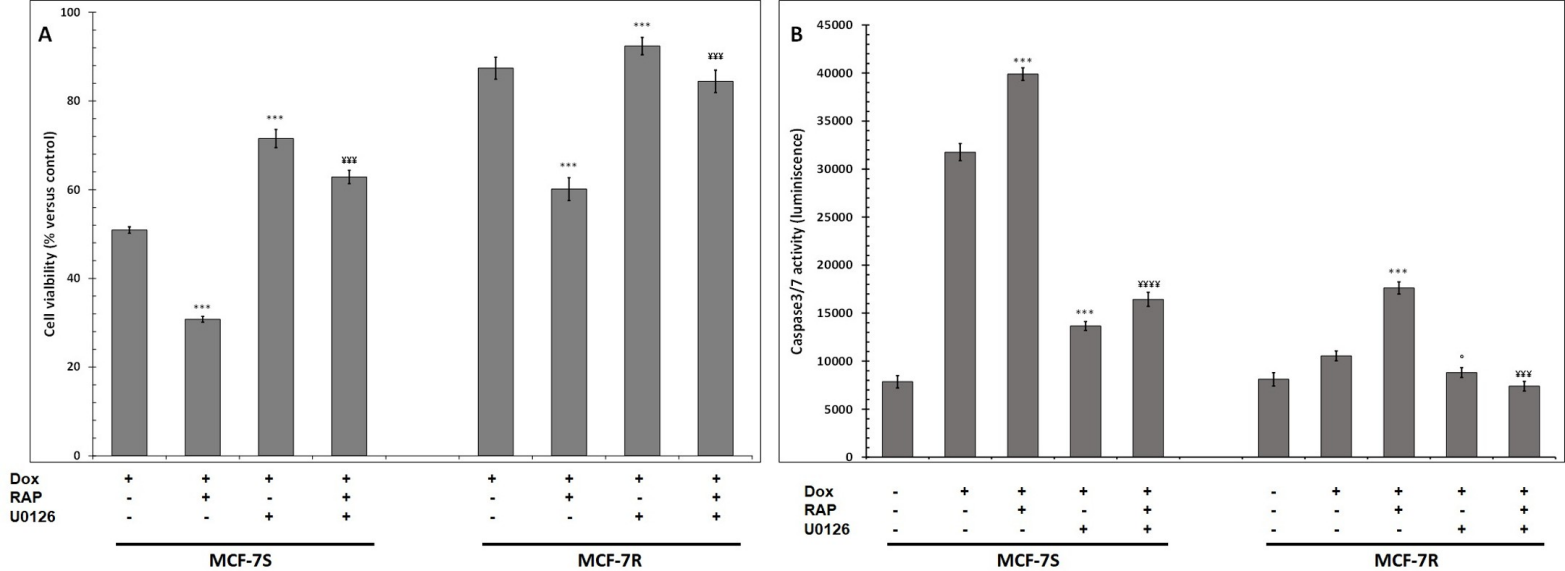
ERK1/2 inhibition reduced Dox cytotoxic effects on MCF-7 cells. MCF-7S and MCF-7R cells were incubated with 1 μM Dox with or without 500 nM RAP and 1 μM U0126 48 hours. **A**, Cell viability was measured using UptiBlue Viable Cell Assay. **B**, Caspase-7 activity was measured by caspACE assay kit. The results obtained from three independent experiments (% of control) were represented with standard deviation (S.D.). Student’s t-test was used for the statistical significance of different values. *** p<0.001 compared to Dox-treated cells and ¥¥¥ p<0.001, ¥¥¥¥ p<0.0001 compared to Dox- and RAP-treated cells.° NS, U0126-treated cells versus Dox-treated cells.

To determine a possible relationship between ERK1/2 phosphorylation and MCF-7 cell apoptosis, we treated cells with U-0126, a cell permeable ERK1/2 signalling pathway inhibitor and analysed cell viability. As shown in Fig 10, U-0126 prevented Dox-induced apoptosis in MCF-7 cells and inhibited RAP-sensitized cell to Dox treatment. Collectively, our data support the hypothesis that ERK1/2 activation is essential for Dox mediated effects in MCF-7 cells.

## 4. Discussion

Multidrug-resistance (MDR) is a major obstacle to successful cancer chemotherapy and one important mechanism of MDR involves the plasma membrane glycoprotein P-gp. This transporter confers to cancer cells the ability to resist to cytotoxic drugs by pumping the drug out of the cells and thus reducing its cytotoxicity [21, 54, 55]. Several studies have demonstrated a negative correlation between P-gp expression levels and chemosensitivity in a range of human malignancies [55–57]. It is well established that P-gp actively effluxes cytotoxic substrates such as Dox [22]. Many studies showed that P-gp may traffic in cells through an intracellular endosomal system with protein vesicles transported to endosome compartments and thus forming an intracellular pool [51]. Indeed, P-gp has been reported to bound to the lysosomal membrane thus promoting the accumulation of the chemotherapeutic agent in acidic organelles [58]. This strongly suggested the existence of both spatial and temporal distribution of P-gp and an intracellular traffic between the plasma membrane and endocytic organelles. Our study revealed that P-gp was localized both in and out late endosomes in MCF-7 human breast cancer cells. The localization of P-gp in endosomes has been suggested to serve as an intra-cellular reservoir to P-gp moving to the plasma membrane [17–19] and P-gp was internalized into cells from the plasma membrane to lysosomes via the endocytic pathway [59]. However, the detailed mechanisms of this P-gp intracellular traffic remain unclear.

LRP-1 is a large multi-functional receptor that regulates endocytosis of numerous ligands and transduces several signaling pathways. LRP-1 is detected in most tissues and highly expressed in liver, brain and lung [60]. In our study, we showed that the MCF-7 human breast cancer cells resistant to Dox expressed a high level of LRP-1 as compared to that of parental non-resistant cells. A higher LRP-1 expression has also been reported in MDA-MB-231 breast cancer cells and HCT-15 colorectal cancer cells [59] and can be related to the pro-tumoral effect of LRP-1 in some types of cancer cells. Concurrently, we observed that MCF-7R cells contained high density lysosomes compared to sensitive cells. LRP-1 has been shown to be essential for the recruitment of molecular adapters and signaling proteins for the initiation of endocytosis [61]. Thus, in our study, we observed an increase of lysosomes characterized by an elevated LAMP-1 level in MCF-7R cells. This result agreed with Dehay et al. studies (2010) showing a decrease of LAMP-1 level corresponded to a reduction of lysosomes number [62]. LAMP-1 was a major constituent of the lysosomal membrane and contributed to protection of the lysosomal membrane and its proteins from the hostile constituents such as hydrogen ion and proteases [63]. Indeed, downregulation of LAMP-1 has been previously shown to sensitize cells to lysosomal mediated death pathways [64] while increased levels of LAMP-1 mRNAs was observed in various human cancers [65–67]. This finding suggested a possible contribution of lysosomes in MCF-7R cell chemoresistance. LRP-1 blockade either by masking ligand binding sites with RAP or by silencing its expression by siRNA interference, reduced the density lysosomes and LAMP-1 level in MCF-7-R cells and sensitized them to Dox treatment. This hypothesis was supported by studies showing implicated lysosomes in drug resistance phenotypes and LAMP-1 in cancer metastasis [66, 68–70]. Several approaches have recently been developed to overcome drug resistance, including reduction of lysosome density by photo-destruction and by permeabilization of the lysosomal membrane or downregulation of LAMP-1 [66, 71].

In parallel, we showed that MCF-7R cells also overexpressed P-gp with a high level in lysosomes, suggesting that LRP-1 might lead to internalization of P-gp through an endocytic pathway. Indeed, LRP-1 blockade or silencing by siRNA reduced both P-gp expression and distribution in MCF-7R cells thus leading to increase Dox nuclear accumulation and therefore cell sensitization to Dox. lysosomal P-gp reduction did not lead to an elevation of its level in other cellular compartments such as the plasma membrane. Indeed, we showed a decrease in the global level of P-gp and also of LAMP-1. Several studies showed that LAMP-1 may protect lysosomal membrane proteins from degradation by proteases and hydrolases [72–75]. In our study, the decrease of LAMP-1 suggested the removal of this protection which could lead to the degradation of P-gp and consequently a decrease of its level at the cellular level. This would support the hypothesis that LAMP-1 was involved in the modulation of P-gp-induced Dox resistance in MCF-7R cells.

Dox-induced apoptosis has been largely described as involving activation of the MAP kinases ERK 1/2 and the caspase 3 in different cell types. In this study, we showed that, in MCF-7 parental cells, Dox involved ERK 1/2 phosphorylation in apoptosis induction. In fact, ERK 1/2 inhibition markedly suppressed the Dox pro-apoptotic effect. Several studies confirmed the implication of ERK1/2 apoptosis activation via the enhancement of Fas ligand expression and the inactivation of BAD, a member of the pro-apoptotic bcl-2 family [76, 77].

Interestingly, in MCF-7 resistant cells characterized by a high P-gp level expression, ERK 1/2 are not activated leading to a resistance to Dox. LRP-1 blockade promoted ERK 1/2 activation and sensitized MCF-7 resistant cells to Dox.

The only publication showing a link between endosomes and ERK1/2 via the p14/MP1 complex was reported by Teis et al. [78]. In MCF-7 cells, MP1 was not detected by Western blot.

**Fig 11:**
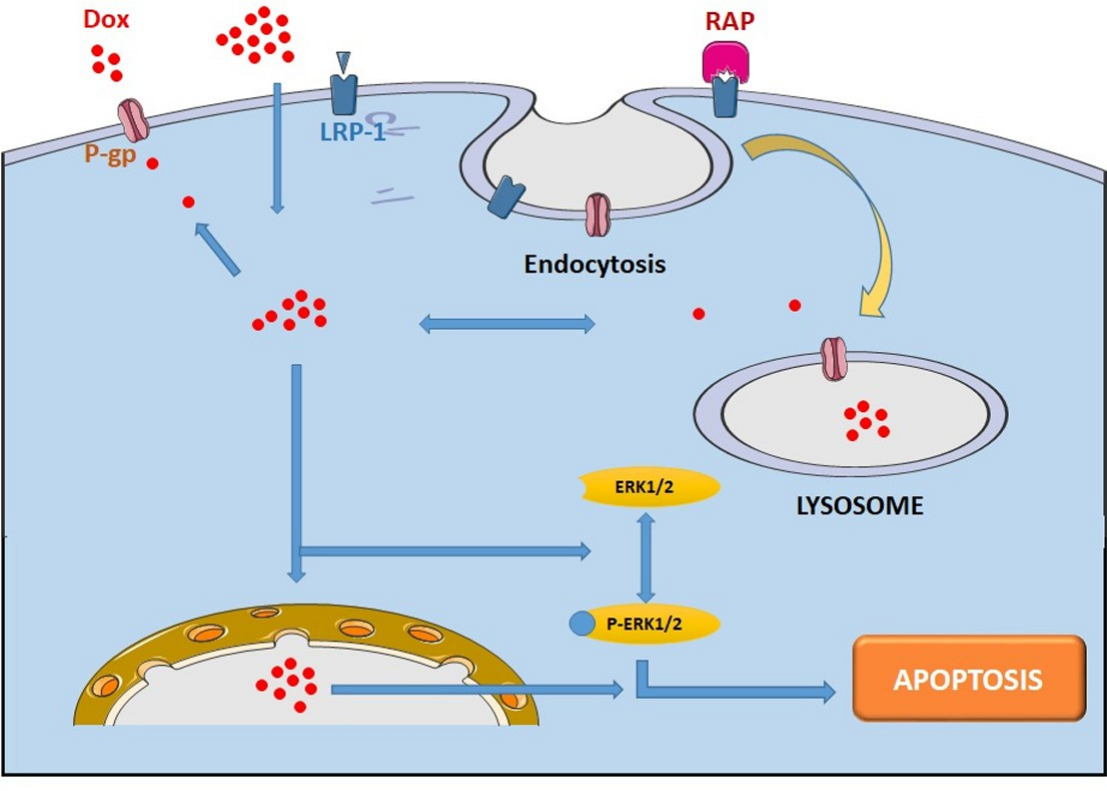
Schematic diagram of P-gp internalization by LRP-1 receptor and its implication in MCF-7 cells drug resistance.

Thus, our data suggest that LRP-1 is able to modulate ERK1/2 signaling pathway by regulating expression and subcellular redistribution of P-gp and by potentializing the P-gp-acquired Dox resistance Fig 11.

Some previous studies have indicated that several survival-related or death-associated signaling pathways, including the PI-3K/Akt and MAPKs pathways, play pivotal roles in the regulation of DNA damage-induced apoptosis [79–83]. Thus, pharmacological modulation of MAPK signaling has been determined to influence apoptotic responses to these anti-cancer drugs. However, the role played by MAPKs depends on cell type and drug concentration [84]. Activation of the ERK 1/2 pathway is generally associated to cell proliferation and/or survival. However, the ERK1/2 pathway can also stimulate apoptosis [85–87].

Our study showed that P-gp was present in both plasma membrane and lysosomes of MCF-7 resistant cells. P-gp internalization was controlled by LRP-1 *via* an endocytic traffic, suggesting that LRP-1 contributed to lysosomal trafficking of P-gp and consequently controlled the P-gp acquired-resistance of MCF-7 cells to Dox. Previous studies have confirmed that P-gp is functional on the plasma membrane and in lysosomes. Lysosomal P-gp increased the trapping of Dox, preventing it from reaching its targets, and conferred drug resistance. This resistance can be circumvented by lysosomotropic agents that can induce increased Dox cytotoxicity. [20, 88].

While the canonical mechanism of P-gp action is mediated by its localization and activity at the plasma membrane, the role of intracellular P-gp may be crucial to consider in drug therapy [68]. Our study reported that intracellular P-gp-mediated sequestration of the drug in lysosomes could serve as an additional mechanism to drug efflux *via* plasma membrane-bound P-gp for multidrug-resistance. Several studies been demonstrated that P-gp not only functions to efflux drugs out of the cell when present on the plasma membrane but also serves a functional intracellular role in the lysosomal membrane to induce resistance [20]. This mechanism may be exploitable with combination therapy to overcome intracellular MDR. Indeed, classical lysosomotropic agents, such as chloroquine, in combination with current ineffective drug treatments for MDR, could cause the lysosomal-release of these agents to increase their chances of reaching sensitive intracellular targets and regain efficacy [20, 89]. Inhibition of P-gp internalization by blocking LRP-1 could offer a fruitful new strategy to enhance lysosomal accumulation of cytotoxic drugs and serve as a new target and an alternative strategy to effectively battle multidrug-resistance.
